# Supplementary material for: Tobacco smoking clusters in households affected by tuberculosis in an individual participant data meta-analysis of national tuberculosis prevalence surveys: Time for household-wide interventions?
Source: PLOS Glob Public Health. 2024 Feb 29;4(2):e0002596. doi: 10.1371/journal.pgph.0002596 (PMC10903843; doi:10.1371/journal.pgph.0002596)
Supplement: S1 Appendix — (DOCX) [file pgph.0002596.s002.docx]

# S1 Appendix. Search strategy

Medline

| 1 | tuberculosis.m_titl. |
| --- | --- |
| 2 | prevalence.m_titl. |
| 3 | survey.tw. |
| 4 | 1 and 2 and 3 |
| 5 | limit 4 to yr="2000 -Current" |

EMBASE

| 1 | tuberculosis.m_titl. |
| --- | --- |
| 2 | prevalence.m_titl. |
| 3 | survey.ti,ab,kw. |
| 4 | 1 and 2 and 3 |
| 5 | limit 4 to yr="2000 -Current" |
