# Supplementary material for: Tobacco smoking clusters in households affected by tuberculosis in an individual participant data meta-analysis of national tuberculosis prevalence surveys: Time for household-wide interventions?
Source: PLOS Glob Public Health. 2024 Feb 29;4(2):e0002596. doi: 10.1371/journal.pgph.0002596 (PMC10903843; doi:10.1371/journal.pgph.0002596)
Supplement: S2 Appendix — (DOCX) [file pgph.0002596.s003.docx]

# S2 Appendix. Supplementary methods

*Handling of missing data*

Not all outcome variables were collected in all surveys. We performed multiple imputation separately for each outcome by restricting to surveys that collected the outcome. For addressing sporadic missingness in each outcome, we conducted multiple imputation using multilevel fully conditional specifications. The main predictor, TB status, was classified into three groups: people with TB, members of households with TB, and members of households without TB. The imputation models included NCD and their risk factors, TB status, age, gender, TB symptoms, and chest x-ray findings. BMI was included after transformation and then imputed (i.e. so-called 'just another variable' approach).^1^ Because the number of surveys reporting most outcomes was small, the model included fixed intercepts for surveys. Sampling clusters were included as random intercepts.

We generated 20 multiply imputed data sets with 20 iterations between successive imputations. We assessed model convergence visually. All primary analyses were performed across multiply imputed datasets; substantive models were fitted on each imputed dataset, and their outputs were combined using Rubin's rules.

*Sensitivity analysis- quantitative bias analysis*

We conducted a record-level quantitative bias analysis to explore the impact of the misclassification of diabetes and hypertension status.^2^ We assumed various levels of accuracy (i.e. sensitivity and specificity) of diabetes and hypertension status, testing both non-differential and differential misclassification by TB status. Based on the literature, we varied the sensitivity of self-reported diabetes and hypertension between 40% to 80%.^3-6^ The prevalence of self-reported diabetes in the study population was 2.8% in people without TB (see Results). This suggests a high specificity of diabetes in the study population consistent with the literature.^3,4^ For hypertension, we tested a specificity of 85%, 90%, and 95%.^5,6^ We adapted the approach described by Fox et al. while using fixed levels of sensitivity and specificity.^2^ We first sampled one of the 20 multiply imputed datasets and estimated positive and negative predictive values for diabetes/hypertension given their observed status. Second, using the predictive values, we simulated a new variable representing the true diabetes/hypertension status drawing at random from a Bernoulli distribution. We fitted a logistic regression model using the new variable as an outcome and TB status as a predictor, adjusted for age and gender. Finally, to account for random errors, we sampled a standard normal deviate, multiplied it by the standard error of the bias-adjusted association, and combined it with the point estimate from the model. We repeated the above process 1000 times and presented the median and 2.5^th^ and 97.5^th^ percentiles as uncertainty intervals. To reduce the computation time, the regression model excluded random intercepts for clusters unlike the model used in the primary analysis. To compare the results between models that are comparable, we compared the results of this sensitivity analysis with those from the models using observed diabetes and hypertension status without random intercepts for clusters.

**Refereneces**

1. Von Hippel PT. HOW TO IMPUTE INTERACTIONS, SQUARES, AND OTHER TRANSFORMED VARIABLES. *Sociol Methodol* 2009; **39**(1): 265-91.

2. Fox MP, MacLehose RF, Lash TL. Probabilistic Bias Analysis for Simulation of Record-Level Data. Applying Quantitative Bias Analysis to Epidemiologic Data Second Edition. Switzerland: Springer; 2021: 291-326.

3. Ning M, Zhang Q, Yang M. Comparison of self-reported and biomedical data on hypertension and diabetes: findings from the China Health and Retirement Longitudinal Study (CHARLS). *BMJ Open* 2016; **6**(1): e009836.

4. Schneider AL, Pankow JS, Heiss G, Selvin E. Validity and reliability of self-reported diabetes in the Atherosclerosis Risk in Communities Study. *Am J Epidemiol* 2012; **176**(8): 738-43.

5. Gonçalves VSS, Andrade KRC, Carvalho KMB, Silva MT, Pereira MG, Galvao TF. Accuracy of self-reported hypertension: a systematic review and meta-analysis. *J Hypertens* 2018; **36**(5): 970-8.

6. Wellman JL, Holmes B, Hill SY. Accuracy of self-reported hypertension: Effect of age, gender, and history of alcohol dependence. *J Clin Hypertens (Greenwich)* 2020; **22**(5): 842-9.
