# Supplementary material for: Tobacco smoking clusters in households affected by tuberculosis in an individual participant data meta-analysis of national tuberculosis prevalence surveys: Time for household-wide interventions?
Source: PLOS Glob Public Health. 2024 Feb 29;4(2):e0002596. doi: 10.1371/journal.pgph.0002596 (PMC10903843; doi:10.1371/journal.pgph.0002596)
Supplement: S1 Table — (DOCX) [file pgph.0002596.s004.docx]

## S1 Table. List of variables requested.

| **Household level information** |
| --- |
| Cluster ID |
| Household ID |
| Availability of assets (e.g. refrigerator) |
| Access to clean water |
| Use of biomass fuel |
| Number of rooms |
| Household Income |
| Other variables relevant to socioeconomic status collected in surveys. |
| Number of household members |
| Education status |
| **Individual data** |
| Sex |
| Age |
| Household id |
| Smoking |
| Alcohol use |
| HIV status |
| Body weight |
| BMI |
| Occupation |
| Education level |
| Diabetes |
| Hypertension |
| Silicosis |
| COPD |
| Asthma |
| Past history of TB |
| Current TB treatment |
| Symptoms |
| Chest X-ray abnormality |
| Smear microscopy result |
| Xpert MTB/RIF result |
| Sputum Culture result |
