# Supplementary material for: Tobacco smoking clusters in households affected by tuberculosis in an individual participant data meta-analysis of national tuberculosis prevalence surveys: Time for household-wide interventions?
Source: PLOS Glob Public Health. 2024 Feb 29;4(2):e0002596. doi: 10.1371/journal.pgph.0002596 (PMC10903843; doi:10.1371/journal.pgph.0002596)
Supplement: S2 Table — (DOCX) [file pgph.0002596.s005.docx]

## S2 Table. Categorisations of current alcohol drinking by surveys

| Eswatini | None  Once a week  Monthly or less  2-4 times a month  2-3 times a week  4 or more times a week |
| --- | --- |
| Gambia | None  Occasionally 1-2 times/wk 3-5 times/wk > 5 times/wks |
| Ghana | None  Once in past year Once in 6 months Once in a month Once in a week 3-4 times a week Everyday |
| Mongolia | None Once a month or less 2-4 times a month 2-3 times a week At least 4 times a week |
| Mozambique | None  1 times a month or less  2 to 4 times a month  2 to 3 times a week  4 or more times a week |
| Namibia | How many days have you consumed alcohol in the past two weeks?  None  1-2  3-4  5+ |
| South Africa | None  Once a month or less  2- 4 times a month  2-3 times a week  4 or more times a week |
| UR Tanzania | None  Sporadic  Monthly  Weekly  Daily |

Other countries did not collect data on alcohol drinking.
