# Supplementary material for: Tobacco smoking clusters in households affected by tuberculosis in an individual participant data meta-analysis of national tuberculosis prevalence surveys: Time for household-wide interventions?
Source: PLOS Glob Public Health. 2024 Feb 29;4(2):e0002596. doi: 10.1371/journal.pgph.0002596 (PMC10903843; doi:10.1371/journal.pgph.0002596)
Supplement: S3 Table — (DOCX) [file pgph.0002596.s006.docx]

## S3 Table. Characteristics of participants by survey

|  | group | Bangladesh | Eswatini | Gambia | Ghana | Indonesia | Lesotho | Malawi | Mongolia | Mozambique | Namibia | Nigeria | Philippines | South Africa | UR Tanzania | Uganda | Viet Nam |
| --- | --- | --- | --- | --- | --- | --- | --- | --- | --- | --- | --- | --- | --- | --- | --- | --- | --- |
|  | Year | 2015-16 | 2019 | 2012 | 2013 | 2013-14 | 2019 | 2013-14 | 2014-15 | 2017-2020 | 2018 | 2012 | 2016 | 2017-2019 | 2012 | 2014-15 | 2017-18 |
| Age | Median (IQR) years | 33 (23-46) | 32 (21-48) | 28 (20-41) | 35 (23-50) | 37 (26-50) | 37 (24-56) | 30 (21-44) | 39 (28-52) | 29 (20-44) | 34 (23-48) | 32 (23-48) | 37 (24-52) | 37 (25-55) | 35 (23-50) | 29 (21-42) | 47 (33-58) |
|  | N | 98710 | 24358 | 43099 | 61724 | 67942 | 21719 | 31579 | 50309 | 32445 | 29495 | 44186 | 46689 | 35191 | 50418 | 41154 | 61763 |
| Gender | Male, n (%) | 44365 (44.9%) | 9939 (40.8%) | 17504 (40.6%) | 24688 (40.0%) | 31632 (46.6%) | 8597 (39.6%) | 13099 (41.5%) | 20070 (39.9%) | 14001 (43.2%) | 12595 (42.7%) | 18178 (41.1%) | 20893 (44.7%) | 13388 (38.0%) | 20735 (41.1%) | 17485 (42.5%) | 27150 (44.0%) |
|  | N | 98710 | 24358 | 43100 | 61726 | 67944 | 21719 | 31579 | 50309 | 32445 | 29495 | 44186 | 46689 | 35191 | 50436 | 41154 | 61763 |
| Smoking | Current smoker | 21416 (21.7%) | 667 (8.2%) | 4961 (11.5%) | 152 (5.4%) | 23025 (33.9%) | 5848 (27.0%) | 3031 (9.6%) | 12291 (24.5%) | 1337 (10.7%) | 2196 (18.1%) | 2139 (4.8%) | 10749 (23.1%) | 9367 (26.7%) | 875 (14.6%) | 3020 (7.3%) | 1459 (32.2%) |
|  | N | 98710 | 8105 | 43100 | 2819 | 67944 | 21648 | 31579 | 50096 | 12520 | 12112 | 44185 | 46514 | 35117 | 6002 | 41147 | 4532 |
| Alcohol | No drinking, n (%) | NA | 6632 (82.4%) | 42655 (99.0%) | 1856 (65.8%) | NA | NA | NA | 27149 (54.4%) | 10248 (81.7%) | 7086 (67.4%) | NA | NA | 23323 (66.3%) | 3744 (62.5%) | NA | NA |
|  | Weekly or less, n (%) | NA | 991 (12.3%) | 371 ( 0.9%) | 570 (20.2%) | NA | NA | NA | 22616 (45.3%) | 1938 (15.4%) | 2028 (19.3%) | NA | NA | 9858 (28.0%) | 1166 (19.4%) | NA | NA |
|  | Twice per week or more, n (%) | NA | 427 ( 5.3%) | 59 ( 0.1%) | 393 (13.9%) | NA | NA | NA | 129 ( 0.3%) | 362 ( 2.9%) | 1399 (13.3%) | NA | NA | 2010 ( 5.7%) | 1085 (18.1%) | NA | NA |
|  | N | NA | 8050 | 43085 | 2819 | NA | NA | NA | 49894 | 12548 | 10513 | NA | NA | 35191 | 5995 | NA | NA |
| Diabetes | Diabetes, n (%) | NA | 231 (3.8%) | NA | 103 (3.9%) | 1654 (2.4%) | NA | NA | 1235 (2.5%) | NA | 183 (1.5%) | NA | 1866 (4.0%) | 1784 (5.1%) | 61 (1.0%) | NA | 376 (8.3%) |
|  | N | NA | 6005 | NA | 2631 | 67944 | NA | NA | 50305 | NA | 11897 | NA | 46689 | 34651 | 5990 | NA | 4530 |
| HIV | Positive, n (%) | NA | 1674 (30.9%) | NA | NA | NA | 3915 (23.0%) | 1840 (9.3%) | 75 (0.1%) | 2966 (13.0%) | 3338 (13.7%) | NA | NA | 4606 (17.4%) | 307 (5.1%) | 422 (9.6%) | NA |
|  | N | NA | 5415 | NA | NA | NA | 17031 | 19703 | 50306 | 22845 | 24391 | NA | NA | 26406 | 6002 | 4394 | NA |
| Hypertension | Hypertension, n (%) | NA | NA | NA | NA | NA | NA | NA | 19990 (40.4%) | NA | 1851 (15.6%) | NA | NA | NA | NA | NA | NA |
|  | N | NA | NA | NA | NA | NA | NA | NA | 49495 | NA | 11897 | NA | NA | NA | NA | NA | NA |
| BMI | Mean (SD) kg/m^2^ | NA | NA | NA | NA | NA | NA | NA | 26.1 ( 5.0) | NA | 23.1 ( 5.3) | 23.8 ( 4.8) | NA | NA | 21.8 ( 4.2) | NA | NA |
|  | N | NA | NA | NA | NA | NA | NA | NA | 48239 | NA | 10827 | 40673 | NA | NA | 5796 | NA | NA |
| Active TB | Active TB, n (%) | 278 (0.3%) | 70 (0.3%) | 77 (0.2%) | 202 (0.3%) | 426 (0.6%) | 132 (0.6%) | 132 (0.4%) | 248 (0.5%) | 89 (0.3%) | 119 (0.4%) | 233 (0.5%) | 466 (1.1%) | 234 (0.7%) | 159 (0.3%) | 160 (0.4%) | 221 (0.4%) |
|  | N | 98541 | 23331 | 42588 | 61541 | 67625 | 21083 | 31463 | 49496 | 29697 | 27921 | 42766 | 44333 | 33700 | 49485 | 40851 | 61329 |

TB: tuberculosis; IQR’ interquartile range; SD: standard deviation; HIV: human immunodeficiency virus; BMI: body mass index; NA: not available
