# Supplementary material for: Tobacco smoking clusters in households affected by tuberculosis in an individual participant data meta-analysis of national tuberculosis prevalence surveys: Time for household-wide interventions?
Source: PLOS Glob Public Health. 2024 Feb 29;4(2):e0002596. doi: 10.1371/journal.pgph.0002596 (PMC10903843; doi:10.1371/journal.pgph.0002596)
Supplement: S4 Table — (DOCX) [file pgph.0002596.s007.docx]

## S4 Table. Quality of individual surveys

| Survey | Selection | Measurement of the exposure (TB status) | | | | Measurement of the outcomes | | Missing data |
| --- | --- | --- | --- | --- | --- | --- | --- | --- |
|  | # participated/# eligible (%) | Symptom screening criteria | Chest x-ray criteria | | Diagnostic method | Diagnosis of diabetes | Diagnosis of hypertension | NCD data sought in all participants? |
| Bangladesh | 98710/108834 (90.7) | Scoring based on cough, haemoptysis, weight loss, fever, and/or night sweats | | Any lung abnormality | Smear, culture, and Xpert | NA | NA | Yes |
| Eswatini | 24358/NA (NA) | Cough of any duration, fever for ≥ 2 weeks, unexplained weight loss ≥ 2 weeks, and/or night sweats ≥ 2 weeks | | Any lung abnormality | Xpert. Culture on Xpert positive samples | Self-report | NA | In participants eligible for sputum collection and a randomly selected subset of the others. |
| Gambia | 43100/55832 (77.2) | Cough ≥ 2 weeks,  Cough < 2 weeks with ≥ 2 other TB symptoms*, or  No cough with ≥ 3 other TB symptoms* | | Any lung or mediastinum abnormality | Smear and Culture. Xpert for survey TB cases | NA | NA | Yes |
| Ghana | 61726/67757 (91.1) | Cough ≥ 2 weeks | | Any lung abnormality | Smear and Culture. Xpert on smear+ samples, and if cultures were contaminated | Self-report | NA | In participants who had cough ≥ 2 weeks, TB diagnosis, or treatment history |
| Indonesia | 67944/76576 (88.7) | Cough ≥ 2 weeks and/or haemoptysis | | Any lung or pleura abnormality | Smear and Culture. Xpert on smear+ and non-conclusive culture samples | Self-report | NA | Yes |
| Lesotho | 21719/26857 (80.9) | Cough ≥ 2 weeks, fever, weight loss, and/or night sweats | | Any lung abnormality | Xpert and culture | NA | NA | Yes |
| Malawi | 31579/39026 (80.9) | Any symptoms** ≥ 1 week | | Any lung abnormality | Smear and Culture. Xpert on smear+ or if culture contaminated | NA | NA | Yes |
| Mongolia | 50309/60031 (83.8) | Cough ≥ 2 weeks | | Any lung abnormality | Smear and Culture, Xpert on smear+ samples | Self-report | Blood pressure measurement and self-report | Yes |
| Mozambique | 32445/43442 (74.7) | Cough ≥2 weeks, blood in sputum, and/or any cough with one of the five symptoms/signs for ≥ 2 weeks*** | | Any lung or mediastinum abnormality or CAD4TB score ≥ 40 | Smear, Xpert, and Culture | NA | NA | In participants eligible for sputum collection and a randomly selected subset of the others. |
| Namibia | 29495/38353 (76.9) | Cough, night sweats, fever, and/or weight loss | | Any lung abnormality or CAD4TB score ≥60 | Smear, Xpert, and Culture | Self-report | Self-report | In participants eligible for sputum collection and a randomly selected subset of the others. |
| Nigeria | 44186/77707 (56.8) | Cough ≥ 2 weeks | | Any lung abnormality | Smear, culture, and Xpert | NA | NA | Yes |
| Philippines | 35191/53250 (66.1) | Cough ≥2 weeks, blood in the sputum, and/or haemoptysis | | Any lung abnormality | Smear and Culture | Self-report | NA | Yes |
| South Africa | 46689/61466 (76) | Any cough, fever, night sweats, and/or weight loss | | Any TB suggestive abnormality | Xpert Ultra and culture | Self-report | NA | Yes |
| United Republic of Tanzania | 50447/65664 (76.8) | Cough ≥ 2 weeks, haemoptysis, fever  ≥2 weeks, weight loss, and/or night sweats | | Any lung (or mediastinum) abnormality | Smear, culture, and Xpert  A concern raised about the validity of the number of bacteriologically positive cases. | Self-report | NA | In participants eligible for sputum submission |
| Uganda | 41154/45293 (90.9) | Cough ≥ 2 weeks | | Any lung abnormality | Smear and culture. Xpert on smear+ samples | NA | NA | Yes |
| Viet Nam | 61763/87207 (70.8) | Productive cough ≥ 2 weeks | | Any lung abnormality | Smear and culture | Self-report | NA | In participants eligible for sputum submission |

*Chest pain, night sweats, shortness of breath, loss of appetite, weight loss, fever, haemoptysis.

**Cough, sputum production, haemoptysis, chest pain, weight loss, night sweats, fatigue, fever, and shortness of breath.

***Chest pain, unexplained fever, night sweats, weight loss, and low mid-upper arm circumference
