# Supplementary material for: Tobacco smoking clusters in households affected by tuberculosis in an individual participant data meta-analysis of national tuberculosis prevalence surveys: Time for household-wide interventions?
Source: PLOS Glob Public Health. 2024 Feb 29;4(2):e0002596. doi: 10.1371/journal.pgph.0002596 (PMC10903843; doi:10.1371/journal.pgph.0002596)
Supplement: S5 Table — (DOCX) [file pgph.0002596.s008.docx]

S5 Table. Association between NCD or their risk factors in people with TB and those in members of households with TB, adjusted for age and gender of TB patients and household members

|  | **Current smoker** | | **Alcohol drinking twice per week or more** | | | **Diabetes** | | **Hypertension** | | **BMI** | |
| --- | --- | --- | --- | --- | --- | --- | --- | --- | --- | --- | --- |
| **NCD/NCD risk factors in people with TB in the same households** | **OR (95% CI)** | **P value** | | **OR (95% CI)** | **P value** | **OR (95% CI)** | **P value** | **OR (95% CI)** | **P value** | **Difference (95% CI) kg/m^2^** | **P value** |
| Current smoker | 1.74 (1.37- 2.21) | <0.0001 | | - | - | - | - | - | - | - | - |
| Alcohol drinking twice per week or more | - | - | | 1.55 (0.53-4.52) | 0.4254 | - | - | - | - | - | - |
| Diabetes | - | - | | - | - | 0.14 (0.00-763.70) | 0.6478 | - | - | - | - |
| Hypertension | - | - | | - | - | - | - | 1.31 (0.80-2.17) | 0.2865 | - | - |
| BMI per 1 kg/m^2^ increase | - | - | | - | - | - | - | - | - | 0.08 ( 0.01- 0.16) | 0.0292 |

Note: Odds ratios were adjusted for age and gender of both TB patients and their household members. Age and BMI were included in the model as continuous variables. E.g. OR for age per 10-year increase indicates an increase in odds for each 10-year increase in age.

OR: odds ratio; CI: confidence interval; BMI: body mass index
