# Supplementary material for: Tobacco smoking clusters in households affected by tuberculosis in an individual participant data meta-analysis of national tuberculosis prevalence surveys: Time for household-wide interventions?
Source: PLOS Glob Public Health. 2024 Feb 29;4(2):e0002596. doi: 10.1371/journal.pgph.0002596 (PMC10903843; doi:10.1371/journal.pgph.0002596)
Supplement: S6 Table — (DOCX) [file pgph.0002596.s009.docx]

S6 Table. Sensitivity analysis- the association between any alcohol drinking and TB status

|  | Unadjusted model | | Adjusted for age and gender | |
| --- | --- | --- | --- | --- |
| Group | OR (95% CI) | P value | OR (95% CI) | P value |
| Member of households without TB | 1 | - |  |  |
| Members of households with TB | 1.15 (1.03-1.29) | 0.0128 | 1.19 (1.06-1.33) | 0.0036 |

OR: odds ratio; CI: confidence interval; TB: tuberculosis
