# Supplementary material for: Tobacco smoking clusters in households affected by tuberculosis in an individual participant data meta-analysis of national tuberculosis prevalence surveys: Time for household-wide interventions?
Source: PLOS Glob Public Health. 2024 Feb 29;4(2):e0002596. doi: 10.1371/journal.pgph.0002596 (PMC10903843; doi:10.1371/journal.pgph.0002596)
Supplement: S7 Table — (DOCX) [file pgph.0002596.s010.docx]

S7 Table. Sensitivity analysis- prevalence of NCD/NCD risk factors in members of households with TB compared to those without TB

|  | **Current smoker** | | **Alcohol drinking twice per week or more** | | **Diabetes** | | **Hypertension** | | **BMI** | |
| --- | --- | --- | --- | --- | --- | --- | --- | --- | --- | --- |
| Group | OR (95% CI) | P value | OR (95% CI) | P value | OR (95% CI) | P value | OR (95% CI) | P value | Difference in Kg/m^2^ (95% CI) | P value |
| Member of households without TB | 1 | - | 1 | - | 1 | - | 1 | - | - | - |
| Members of households with TB (adjusted for age and gender) | 1.32 ( 1.22- 1.43) | <0.0001 | 1.44 (1.02-2.03) | 0.0392 | 1.00 (0.80-1.25) | 0.9935 | 0.89 (0.71-1.10) | 0.2669 | -0.15 (-0.43; 0.12) | 0.2758 |

Note: Excluding surveys that collected NCD/NCD risk factors only in a subset of participants. Odds ratios were adjusted for age and gender.

NCD: non-communicable diseases; OR: odds ratio; CI: confidence interval; BMI: body mass index
