# Supplementary material for: Tobacco smoking clusters in households affected by tuberculosis in an individual participant data meta-analysis of national tuberculosis prevalence surveys: Time for household-wide interventions?
Source: PLOS Glob Public Health. 2024 Feb 29;4(2):e0002596. doi: 10.1371/journal.pgph.0002596 (PMC10903843; doi:10.1371/journal.pgph.0002596)
Supplement: S8 Table — (DOCX) [file pgph.0002596.s011.docx]

## S8 Table. Sensitivity analysis- Association between NCD or their risk factors in people with TB and those in members of households with TB, adjusted for age and gender of TB patients

|  | **Current smoker** | | **Alcohol drinking twice per week or more** | | **Diabetes** | | | **Hypertension** | | | **BMI** | | |
| --- | --- | --- | --- | --- | --- | --- | --- | --- | --- | --- | --- | --- | --- |
| **NCD/NCD risk factors in people with TB in the same households** | **OR (95% CI)** | **P value** | **OR (95% CI)** | **P value** | **OR (95% CI)** | **P value** | **OR (95% CI)** | | **P value** | **Difference in Kg/m^2^ (95% CI)** | | **P value** |  |
| Current smoker | 1.52 (1.27-1.83) | <0.0001 |  |  |  |  |  | |  |  | |  |  |
| Alcohol drinking twice per week or more | - | - | 7.01 (0.39-127.38) | 0.1879 | - | - | - | | - | - | | - |  |
| Diabetes | - | - | - | - | 0.15 (0.00-1078.56) | 0.6676 | - | | - | - | | - |  |
| Hypertension | - | - | - | - | - | - | 1.32 (0.82-2.13) | | 0.2562 | - | | - |  |
| BMI per 1 kg/m^2^ increase | - | - | - | - | - | - | - | | - | 0.10 (0.02-0.18) | | 0.0102 |  |

Note: Odds ratios were adjusted for age and gender of TB patients in the same households. Age and BMI were included in the model as continuous variables.

NCD: non-communicable diseases; OR: odds ratio; CI: confidence interval; BMI: body mass index
