# Supplementary figures and images for: Tobacco smoking clusters in households affected by tuberculosis in an individual participant data meta-analysis of national tuberculosis prevalence surveys: Time for household-wide interventions?
Source: PLOS Glob Public Health. 2024 Feb 29;4(2):e0002596. doi: 10.1371/journal.pgph.0002596 (PMC10903843; doi:10.1371/journal.pgph.0002596)

## S1 Fig. Results of the updated literature search


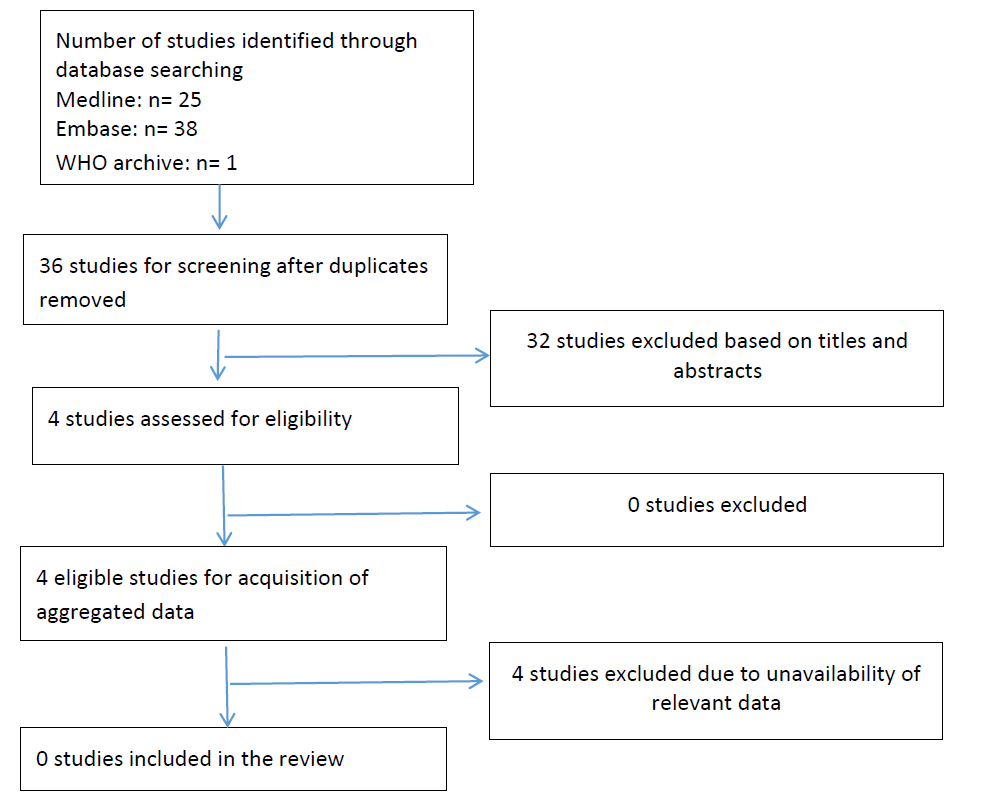


WHO: World Health Organization

Supplement: S1 Fig — (DOCX) [file pgph.0002596.s013.docx]
