# Supplementary material for: Tobacco smoking clusters in households affected by tuberculosis in an individual participant data meta-analysis of national tuberculosis prevalence surveys: Time for household-wide interventions?
Source: PLOS Glob Public Health. 2024 Feb 29;4(2):e0002596. doi: 10.1371/journal.pgph.0002596 (PMC10903843; doi:10.1371/journal.pgph.0002596)
Supplement: S4 Fig — (DOCX) [file pgph.0002596.s016.docx]

## S4 Fig. Current smoking in members of households with TB compared to those without TB, by region


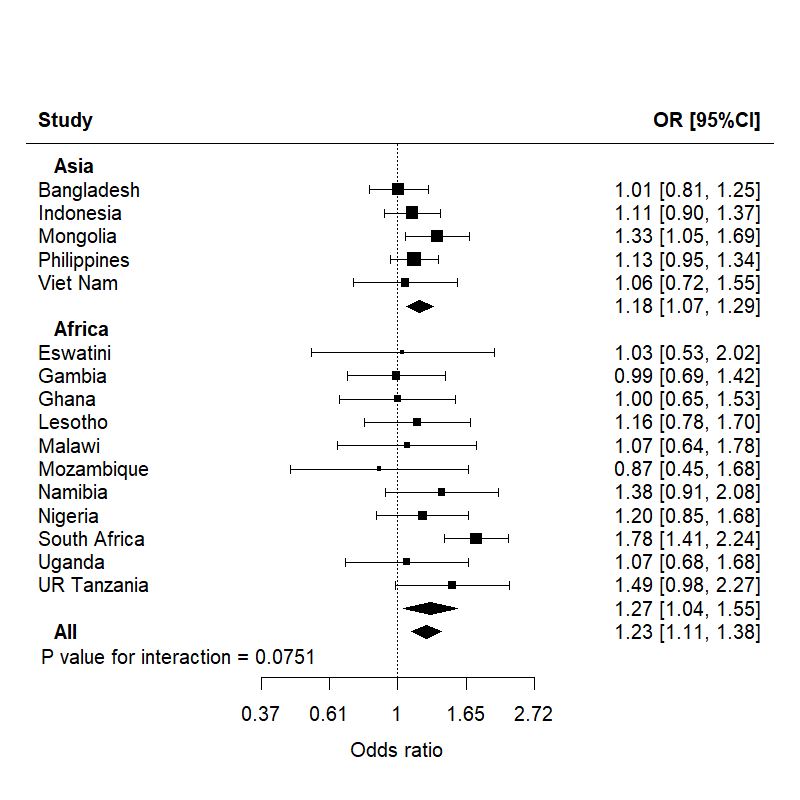


The pooled estimates are based on one-stage meta-analysis.
