# Supplementary material for: Tobacco smoking clusters in households affected by tuberculosis in an individual participant data meta-analysis of national tuberculosis prevalence surveys: Time for household-wide interventions?
Source: PLOS Glob Public Health. 2024 Feb 29;4(2):e0002596. doi: 10.1371/journal.pgph.0002596 (PMC10903843; doi:10.1371/journal.pgph.0002596)
Supplement: S5 Fig — (DOCX) [file pgph.0002596.s017.docx]

## S5 Fig. Alcohol drinking twice per week or more in members of households with TB compared to those without TB


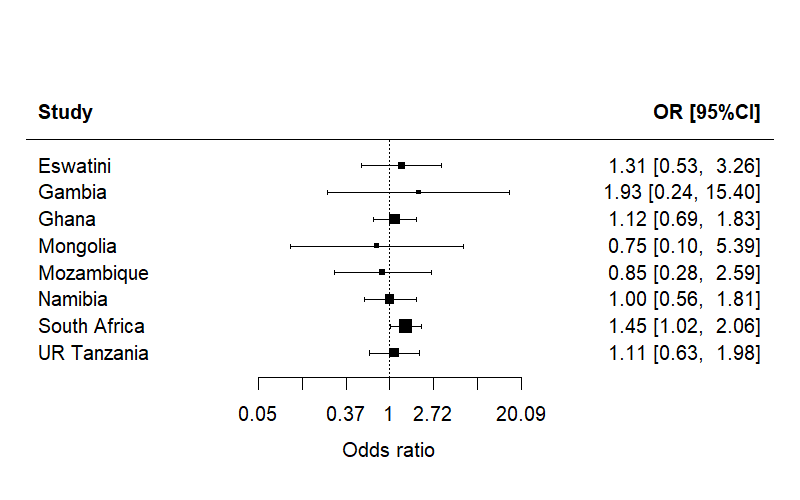


TB: tuberculosis; OR: odds ratio; CI: 95% confidence interval

I-squared=0%, p=0.93, tau^2^=0

Estimates were adjusted for age and gender of participants.
