# Supplementary material for: Tobacco smoking clusters in households affected by tuberculosis in an individual participant data meta-analysis of national tuberculosis prevalence surveys: Time for household-wide interventions?
Source: PLOS Glob Public Health. 2024 Feb 29;4(2):e0002596. doi: 10.1371/journal.pgph.0002596 (PMC10903843; doi:10.1371/journal.pgph.0002596)
Supplement: S8 Fig — (DOCX) [file pgph.0002596.s020.docx]

## S8 Fig. BMI in members of households with TB compared to those without TB


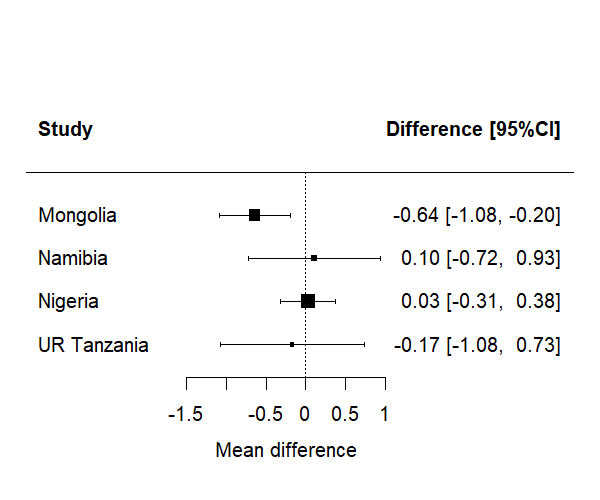


TB: tuberculosis; CI: 95% confidence interval; BMI: body mass index

I-squared=50.4%, p=0.11, tau^2^=0.08

Estimates were adjusted for age and gender of participants.
