# Supplementary material for: Tobacco smoking clusters in households affected by tuberculosis in an individual participant data meta-analysis of national tuberculosis prevalence surveys: Time for household-wide interventions?
Source: PLOS Glob Public Health. 2024 Feb 29;4(2):e0002596. doi: 10.1371/journal.pgph.0002596 (PMC10903843; doi:10.1371/journal.pgph.0002596)
Supplement: S9 Fig — (DOCX) [file pgph.0002596.s021.docx]

## S9 Fig. Association between current smoking of people with TB and the same in their household members


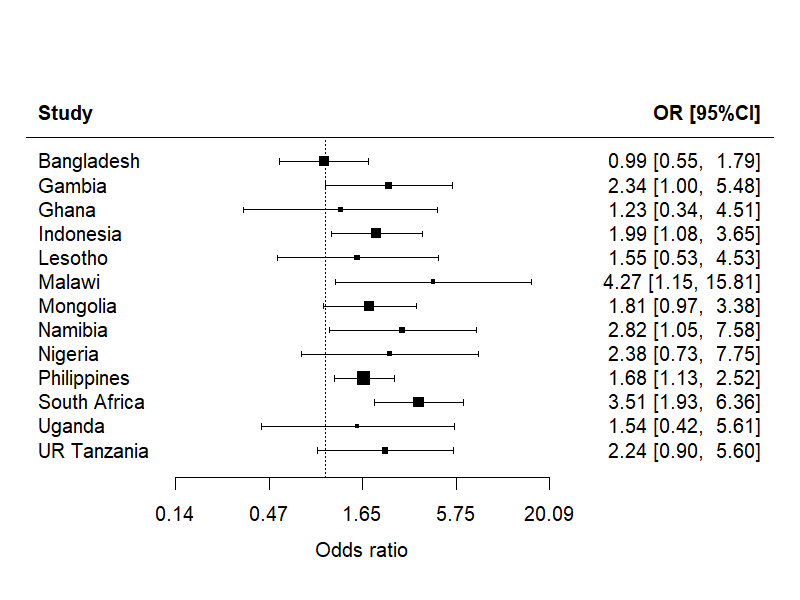


TB: tuberculosis; OR: odds ratio; CI: 95% confidence interval

Note: Estimates were adjusted for age and gender of both people with TB and household members themselves.

Eswatini and Mozambique are not presented due to extremely wide CI, ranging from 0 to > 100000.

I-squared=0% (95% CI 0-53.6), p=0.54, tau^2^=0.04
