# Supplementary material for: Tobacco smoking clusters in households affected by tuberculosis in an individual participant data meta-analysis of national tuberculosis prevalence surveys: Time for household-wide interventions?
Source: PLOS Glob Public Health. 2024 Feb 29;4(2):e0002596. doi: 10.1371/journal.pgph.0002596 (PMC10903843; doi:10.1371/journal.pgph.0002596)
Supplement: S10 Fig — (DOCX) [file pgph.0002596.s022.docx]

## S10 Fig. Association between alcohol drinking of people with TB and the same in their household members


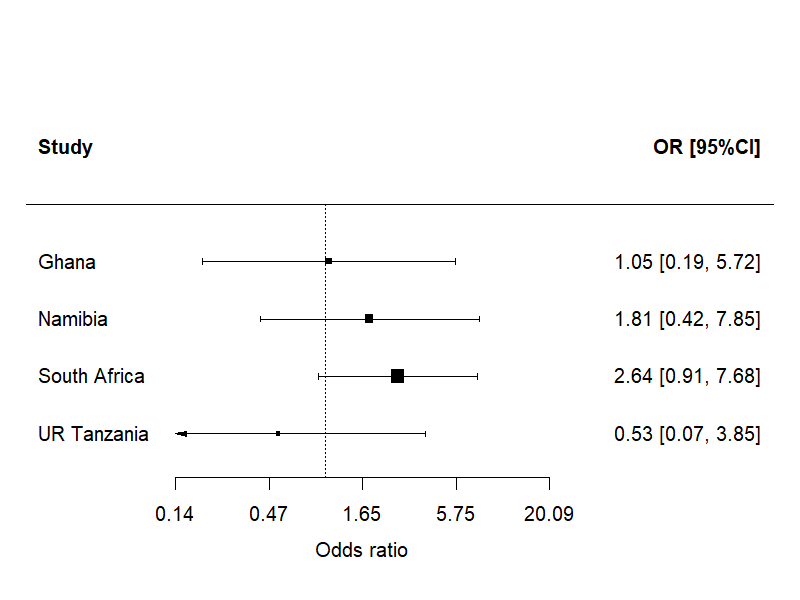


TB: tuberculosis; OR: odds ratio; CI: 95% confidence interval

Note: Estimates were adjusted for age and gender of both people with TB and household members themselves.

Studies are not presented in the plot when the model failed to converge or standard errors were extremely large, resulting in confidence intervals ranging from zero to infinity.

I-squared=0% (95% CI 0-79.2), p=0.68, tau^2^=0
