# Supplementary material for: Tobacco smoking clusters in households affected by tuberculosis in an individual participant data meta-analysis of national tuberculosis prevalence surveys: Time for household-wide interventions?
Source: PLOS Glob Public Health. 2024 Feb 29;4(2):e0002596. doi: 10.1371/journal.pgph.0002596 (PMC10903843; doi:10.1371/journal.pgph.0002596)
Supplement: S12 Fig — (DOCX) [file pgph.0002596.s024.docx]

## S12 Fig. Association between hypertension of people with TB and the same in their household members


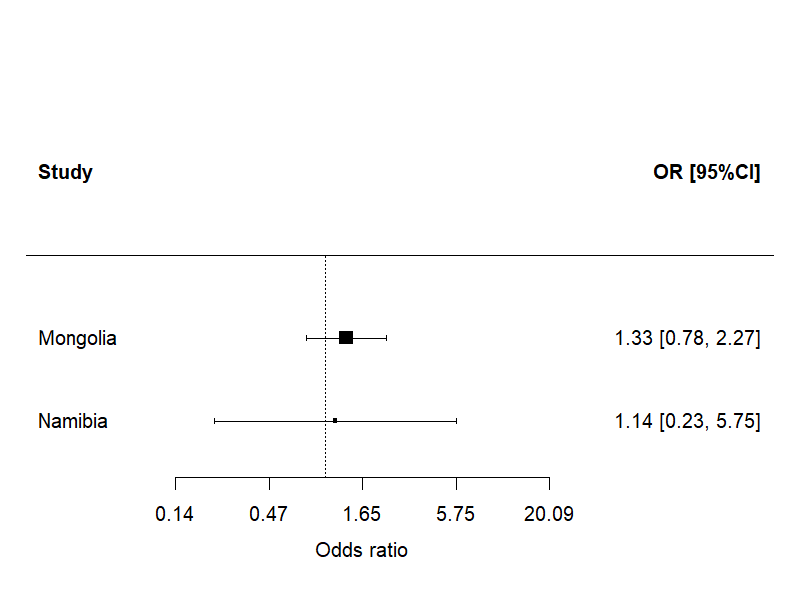


TB: tuberculosis; OR: odds ratio; CI: 95% confidence interval

Note: Estimates were adjusted for age and gender of both people with TB and household members themselves.

Only two studies reported data on hypertension; hence, between-study heterogeneity was not able to estimate.
