# Supplementary material for: Tobacco smoking clusters in households affected by tuberculosis in an individual participant data meta-analysis of national tuberculosis prevalence surveys: Time for household-wide interventions?
Source: PLOS Glob Public Health. 2024 Feb 29;4(2):e0002596. doi: 10.1371/journal.pgph.0002596 (PMC10903843; doi:10.1371/journal.pgph.0002596)
Supplement: S13 Fig — (DOCX) [file pgph.0002596.s025.docx]

## S13 Fig. Association between BMI of people with TB and the same in their household members


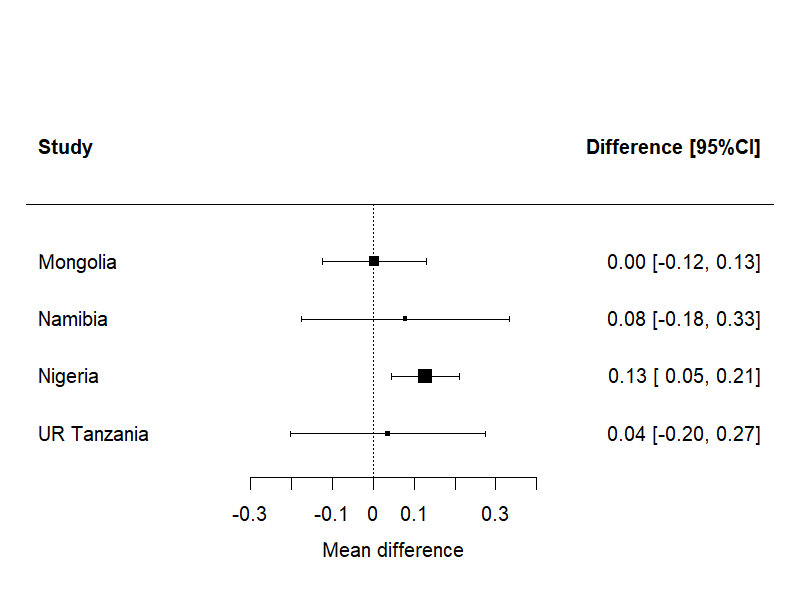


TB: tuberculosis; BMI: body mass index; CI: 95% confidence interval

Note: Estimates were adjusted for age and gender of both people with TB and household members themselves.

I-squared=0% (95% CI 0-84.7), p=0.43, tau^2^=0
