# Supplementary material for: Tobacco smoking clusters in households affected by tuberculosis in an individual participant data meta-analysis of national tuberculosis prevalence surveys: Time for household-wide interventions?
Source: PLOS Glob Public Health. 2024 Feb 29;4(2):e0002596. doi: 10.1371/journal.pgph.0002596 (PMC10903843; doi:10.1371/journal.pgph.0002596)
Supplement: S14 Fig — (DOCX) [file pgph.0002596.s026.docx]

## S14 Fig. Sensitivity analysis-impact of misclassification of diabetes on its association with members of households with TB


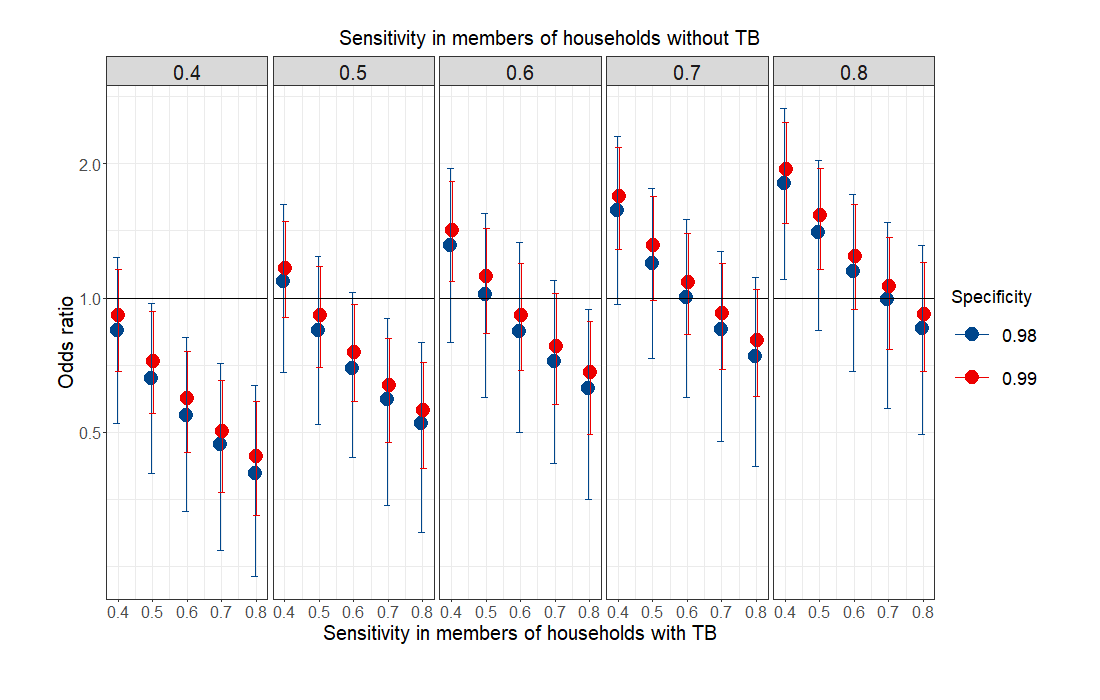


Odds ratios are adjusted for age and gender.

Odds ratios in the analysis using original diabetic status: OR 0.95 (95% CI 0.78-1.17)

The figure presents how the true association between diabetes and being a member of households with TB changes depending on the accuracy of self-reported diabetes. The uncertainty intervals are wide and mostly overlap with null. The direction of the association is driven by the direction and the extent of differential misclassification.
