# Supplementary material for: Tobacco smoking clusters in households affected by tuberculosis in an individual participant data meta-analysis of national tuberculosis prevalence surveys: Time for household-wide interventions?
Source: PLOS Glob Public Health. 2024 Feb 29;4(2):e0002596. doi: 10.1371/journal.pgph.0002596 (PMC10903843; doi:10.1371/journal.pgph.0002596)
Supplement: S15 Fig — (DOCX) [file pgph.0002596.s027.docx]

## S15 Fig. Sensitivity analysis-impact of misclassification of hypertension on its association with members of households with TB


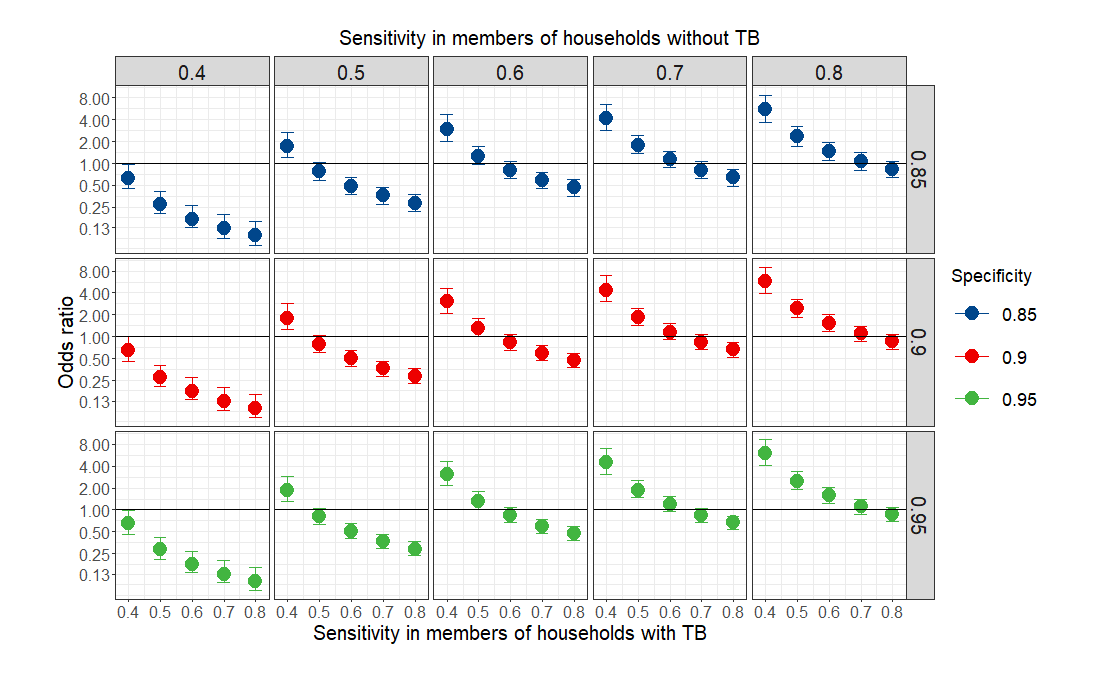


Odds ratios are adjusted for age and gender.

Odds ratios in the analysis using original hypertension status: 0.88 (0.74-1.06)

The figure presents how the true association between hypertension and being a member of households with TB changes depending on the accuracy of the hypertension status in surveys. Overall, when the sensitivity and specificity of hypertension are the same between members of households with TB and those without TB (i.e. non-differential misclassification), the odds are close to null, with uncertainty intervals overlapping with one. The direction of the true association heavily depends on the direction and the magnitude of the differential misclassification.
