# Supplementary material for: Tobacco smoking clusters in households affected by tuberculosis in an individual participant data meta-analysis of national tuberculosis prevalence surveys: Time for household-wide interventions?
Source: PLOS Glob Public Health. 2024 Feb 29;4(2):e0002596. doi: 10.1371/journal.pgph.0002596 (PMC10903843; doi:10.1371/journal.pgph.0002596)
Supplement: Attachment — Submitted filename: Rebutal letter.docx [file pgph.0002596.s028.docx]

Thank you for the opportunity to revise our manuscript. We have revised our manuscript based on the feedback from the reviewer. Please find our response to the reviewer’s comments below.

Reviewer #1: This study evaluates clustering of non-communicable diseases and risk factors among households of people with TB as compared to households without TB using data from TB prevalence surveys. The authors find that people from households affected by TB are more likely to be smokers. Of note is that surveys did not consistently collect data on NCD and risk factors and for some NCDs (hypertension, diabetes) self-reporting may have underestimated their prevalence. This highlights the importance of collecting data on NCD during TB prevalence surveys to better understand burden and improve referral and management for individuals in households affected by TB. The article is very well written, analysed and interpreted and makes a compelling case for integrating NCD data collection within surveys.
line 191 provide in the supplement (methods) additional details on how quality was assessed
were there any geographic/regional differences in smoking clustering

*Response:*

*We thank the reviewer for positive feedback on our manuscript. We appreciate their recognition of the importance of our findings and the quality of our analysis and writing.*

*First, we agree with the reviewer that the self-reporting of NCD was a limitation of data available from the surveys. The availability of individual data allowed us to conduct a quantitative bias analysis, which showed the under-detection of NCD made the findings inconclusive, as the reviewer rightly points out.* ***Thus, as the reviewer suggests, we have suggested standardized data collection in future surveys as follows:***

*Line 363: “Future surveys should consider integrating standardized NCD data collection, such as the WHO STEPwise approach to allow robust analysis of the interaction between NCD and people and households affected by TB [41]”*

***Second, based on the comment from the review, we have conducted an analysis by region and added the following text: “The higher prevalence of smoking among members of households with TB was observed both in Asian and African countries, and there was no significant difference by region (p = 0.0751) (S4 Figure).”***
